# Utidelone inhibits growth of colorectal cancer cells through ROS/JNK signaling pathway

**DOI:** 10.1038/s41419-021-03619-6

**Published:** 2021-04-01

**Authors:** Fuli Li, Tinglei Huang, Yao Tang, Qingli Li, Jianzheng Wang, Xiaojiao Cheng, Wenhui Zhang, Baiwen Zhang, Cong Zhou, Shuiping Tu

**Affiliations:** 1 State Key Laboratory of Oncogenes and related Genes, Department of Oncology, Renji Hospital, School of Medicine, Shanghai Jiao Tong University, Shanghai, 200127 China; 2 Department of Oncology, the Affiliated Cancer Hospital of Zhengzhou University, Henan Cancer Hospital, NO.127, Dongming Road, Zhengzhou, 450008 China; 3 Shanghai Institute of Precision Medicine, Shanghai, 200125 China

**Keywords:** Drug development, Preclinical research

## Abstract

Utidelone (UTD1), a novel microtubule stabilizing agent, is an epothilone B analogue which was produced by genetic engineering. UTD1 has exhibited broad antitumor activity in multiple solid tumors. However, its activity and mechanism in colorectal cancer (CRC) remain to be studied. In this study, UTD1 dramatically inhibited CRC cell proliferation (with 0.38 µg/ml, 0.77 µg/ml IC50 in RKO and HCT116, respectively) in vitro. Immunofluorescence staining showed that UTD1 induced the formation of microtubule bundling and asters in RKO cells. Flow cytometry analysis demonstrated that UTD1 induced cell cycle to arrest in G2/M phase, subsequent apoptosis. Significantly, UTD1 exhibited stronger effect on inducing apoptosis than paclitaxel and 5-FU, especially in HCT15 cells which is ABCB1 high-expression. UTD1 exposure cleaved caspase-3 and poly ADP-ribose polymerase (PARP), decreased mitochondrial membrane potential, released cytochrome c, increased the production of active oxygen and activated c-Jun N-terminal kinase (JNK), suggesting ROS/JNK pathway was involved in this process. Moreover, UTD1 inhibited tumor growth and was more effective and safer compared with paclitaxel and 5-FU in RKO xenograft in nude mice. Taken together, our findings first indicate that UDT1 inhibits tumor growth in CRC xenograft model and may be a promising agent for CRC treatment.

## Introduction

Colorectal cancer (CRC) is one of the most common tumors in the world. More than 2 million people are diagnosed with CRC every year^1,2^. Treatments for CRC include chemotherapies, endoscopic and surgical excision, targeted therapies, local ablative therapies, and immunotherapies^3–6^. Although there are multiple treatments, CRC still is the leading cause of cancer-related deaths^7,8^. Among various treatments, chemotherapies occupy an important role in advanced colorectal cancer, but chemotherapy drugs available in colorectal cancer are limited. Consequently, there remains a significant medical need to find new drugs for CRC.

Antimitotic drugs, also called microtubule targeted drugs, are classified as microtubule-stabilizing or microtubule-destabilizing agents according to their influence on microtubule dynamics. Microtubule-stabilizing agents include taxanes and epothilones. Conversely, vincristine, colchicine, and maytansine belong to microtubule-destabilizing agents^9–12^. Taxanes are approved for various solid tumors such as breast cancer, ovarian cancer, and non-small cell lung cancer^13,14^. Epothilones, which involve epothilone A, B, C, D, E, and F, have anticancer activities similar to paclitaxel, and competed with paclitaxel for binding sites on microtubules^15^. There are now a variety of epothilones and its analogues having entered clinical trials. Ixabepilone (BMS-247550) is the only epothilone analogue approved by the Food and Drug Administration^16,17^. Utidelone (UTD1), a novel microtubule stabilizing agent, is an epothilone B analogue. In breast cancer, UTD1 showed significant antitumor activity and has entered phase III clinical trials^18–21^.

It demonstrated that CRC failed to response to paclitaxel in clinical trials because of serious side effects or multidrug resistance^22,23^. Colorectal cancer constitutively overexpressed P-glycoprotein (P-gp), which is encoded by the multidrug resistance gene (MDR1, ABCB1). P-gp can efflux many structurally and functionally diverse chemotherapeutics, leading to resistance. A lot of chemotherapeutics, such as paclitaxel, docetaxel, and doxorubicin are P-gp substrates^24^. However, unlike paclitaxel, epothilones were less influenced by P-gp; this may be one of the reasons why epothilones were effective in paclitaxel-sensitive and -resistant tumors^25,26^. Preclinical studies indicated that epothilones and its analogues had anticancer activity in CRC cells^27–29^. And a phase I clinical study showed that Ixabepilone coadministration with sunitinib had encouraging clinical activity in metastatic CRC patients^30^. In addition, epothilones are with high water solubility and more tolerable toxicity, and are proved to have fewer side effects compared with paclitaxel. These findings suggest that UTD1 might have antitumor activity for CRC. However, antitumor activity of UTD1 in CRC model has not been investigated.

In this study, we determined the effects of UTD1 on CRC in vitro and in vivo. We found that UTD1 significantly inhibited proliferation of CRC cells in a dose- and time-dependent manner. Flow cytometry showed UTD1induced cell cycle to arrest in G2/M phase and followed by apoptosis, which involved ROS/JNK pathway. Furthermore, UTD1 suppressed growth of tumors and was more effective and safer.

## Results

### UTD1 reduced viability and inhibited proliferation of human CRC cells in vitro

**Fig. 1:**
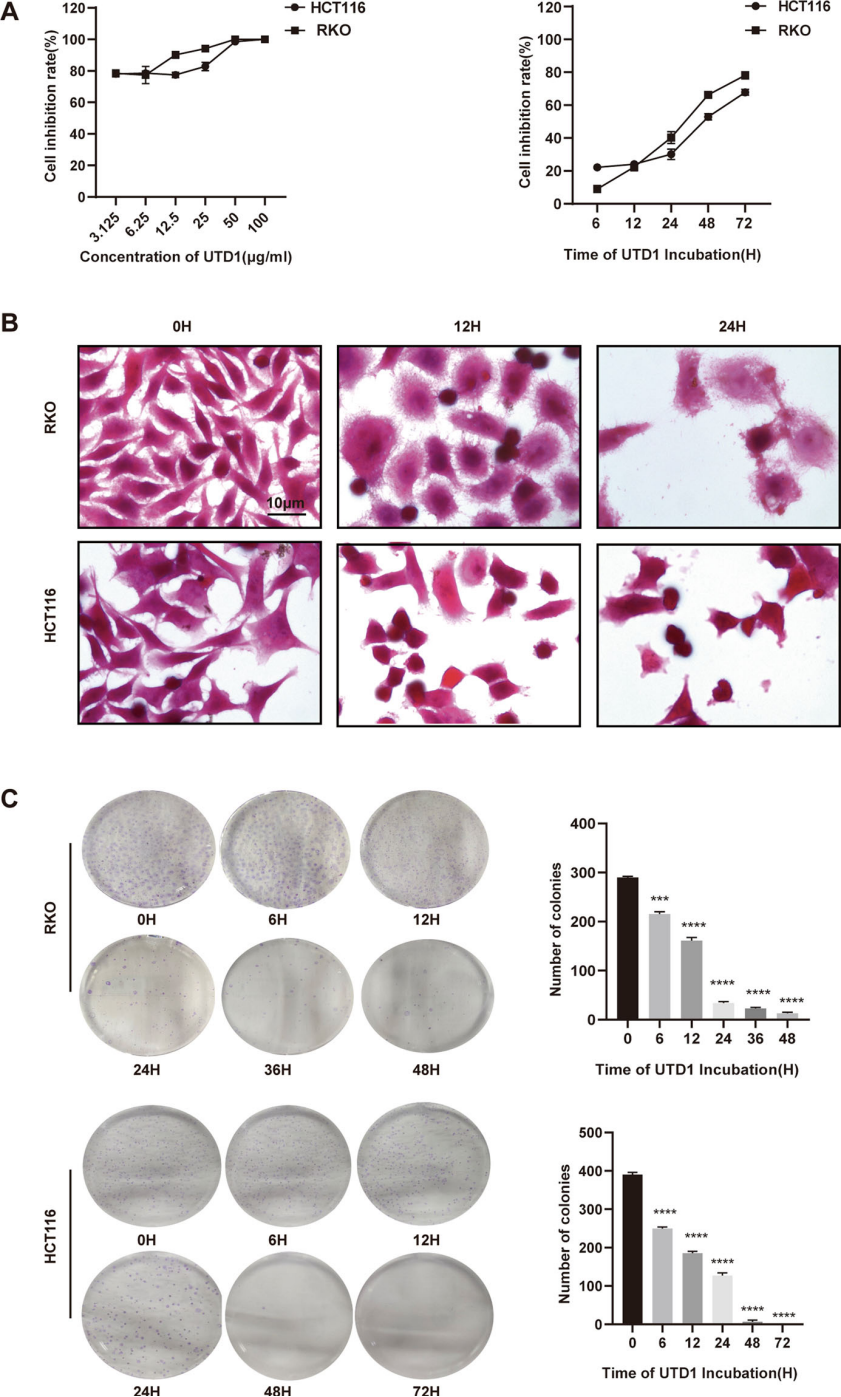
UTD1 inhibited cell viability and proliferation in a dose- and time-dependent manner in CRC cells. **A** Cell viability was measured by CCK-8 after RKO and HCT116 cells were treated with different concentrations of UTD1 for 72 h or 1 µg/ml UTD1 for different time-points. **B** Morphology of cells treated with UTD1 for 0, 12, and 24 h. Scale bar = 10 μm. **C** Colony formation showed UTD1 reduced colony formation of RKO and HCT116 cells. All experiments were performed in triplicate. Results were presented as mean ± SD, ***p < 0.001, ****p < 0.0005 vs. control group.

We first evaluated direct effect of UTD1 on proliferation and survival of CRC cells. UTD1 significantly induced growth inhibition in a dose- and time-dependent manner in RKO and HCT116 cells. After 72 h of incubation, IC50 of RKO and HCT116 cells were 0.38 µg/ml and 0.77 µg/ml, respectively (Fig. 1A). We chose 1 µg/ml UTD1 for further experiments according to IC50 values. To investigate time efficacy, cells were exposed to 1 µg/ml UTD1 for different time-points, inhibition rate of cells increased as incubation was prolonged. Cells exposed to UTD1 exhibited morphological changes, including membrane blebbing, cell shrinkage, nuclear condensation, and fragmentation (Fig. 1B).

To confirm inhibitory effect of UTD1 on cell proliferation, colony formation assay was performed. Consistent with above observations, colony number was reduced in UTD1-treated group in a time-dependent manner (Fig. 1C). These results suggested that UTD1 could suppress proliferation and growth of RKO and HCT116 cells.

### UTD1 changed morphology of tubulin and induced cell cycle to arrest in G2/M phase

**Fig. 2:**
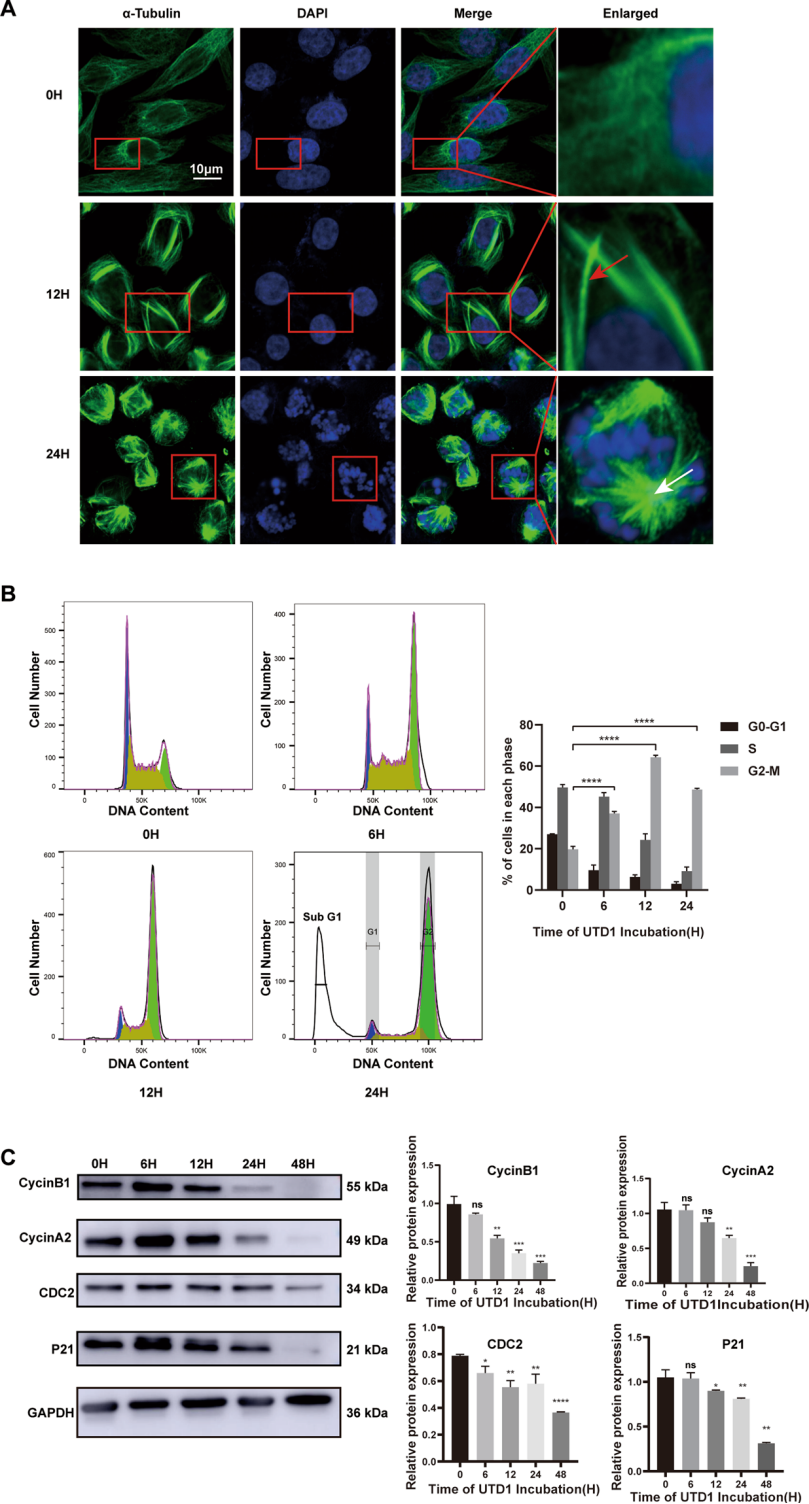
UTD1 changed morphology of tubulin and induced cell cycle to arrest in G2/M phase. **A** Immunofluorescence staining was used to detect morphological change of tubulin after RKO cells were incubated with UTD1. Red and white arrowheads in panels indicate formation of microtubule bundles and asters, respectively. Scale bar = 10 μm. **B** After treatment, RKO cells were fixed, stained, and analyzed. Flow cytometry showed that UTD1 arrested cell cycle in G2/M phase. **C** Proteins involved in G2/M phase were analyzed by Western blotting. All experiments were performed in triplicate. Results were presented as mean ± SD. ns: no statistically significant difference, *p < 0.05, **p < 0.01, ***p < 0.001, ****p < 0.0005 vs. control group.

Because UTD1 is one of the microtubule-stabilizing agents, we investigated the effect of UTD1 on cell microtubule. Immunofluorescence staining showed that UTD1 caused polymerization of microtubule (Fig. 2A), as the formation of polymerized microtubule bundles after 12 h. There were mitotic asters visible after 24 h. To elucidate UTD1’s mode of action, its effect on cell cycle progression was examined. Flow cytometry analysis showed RKO cells arrested in G2/M phase (Fig. 2B), and same results were observed in HCT116 as well as RKO cells which were treated with paclitaxel (Fig. S1A, B). Importantly, more than 60% RKO cells arrested at G2/M phase after being treated with UTD1 after 12 h, while it was less than 40% in the paclitaxel group. To further determine cell cycle arrest in G2/M phase, Western blotting was performed. Consistent with flow cytometry result, proteins mainly appearing in G2/M phase such as cyclinB1, cyclinA2, CDC2, and P21 were decreased (Fig. 2C). These results suggested that UTD1 could change microtubule morphology and block cell cycle at G2/M phase.

### UTD1 induced apoptosis by activating caspase-3 and PARP after G2/M arrest and was more effective than paclitaxel

**Fig. 3:**
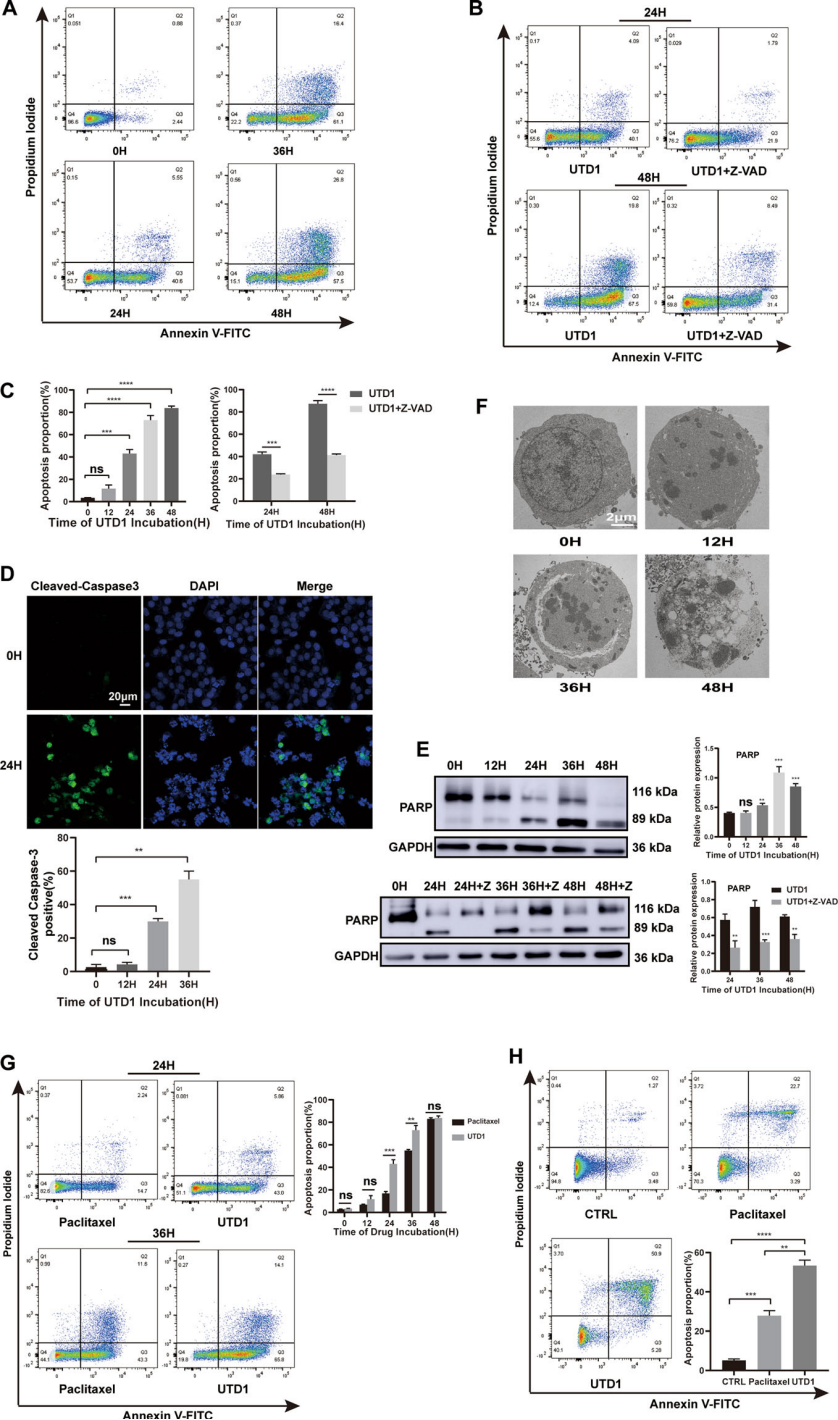
UTD1 induced apoptosis in CRC cells and was more effective than paclitaxel. **A** Flow cytometry using Annexin V-FITC/PI double staining showed UTD1 triggered apoptosis in RKO cells. **B** Cells were preincubated with or without Z-VAD-FMK (50 µm) for 2 h, and then treated with 1 µg/ml UTD1. Apoptosis was detected by flow cytometry. **C** Statistical analysis of **A** and **B**. **D** Cleaved caspase-3 was detected after RKO cells were cultured with UTD1 using immunofluorescence staining. Scale bar = 20 μm. **E** RKO cells were incubated with UTD1 in the presence or absence of 50 μm Z-VAD-FMK. PARP was analyzed by Western blotting. **F** After exposure to UTD1, TEM was used to evaluate apoptosis. Scale bar = 2 μm. **G** After 1 µg/ml paclitaxel or UTD1 treated, flow cytometry was used to detect apoptosis of RKO cells. **H** After exposure to 50 µg/ml UTD1 or paclitaxel 24 h, flow cytometry result showed apoptosis in HCT15 cells. All experiments were performed in triplicate. Results were presented as mean ± SD. ns: no statistically significant difference, **p < 0.01, ***p < 0.001, ****p < 0.0005 vs. control group.

To determine the consequence of UTD1-induced cell-cycle arrest, we evaluated annexin V-FITC/PI staining of treated cells. 48 h later, percentage of apoptosis was approximately 85% in RKO cells (Fig. 3A, C left). Cells treated with UTD1 for 48 h showed drastic increase in apoptosis compared with control group. Pretreatment with a pan-caspase inhibitor, Z-VAD-FMK could reduce apoptosis (Fig. 3B, C right), indicating this apoptotic process required caspase activation. In HCT116 cells, duration of cell arrest was relatively long, and occurrence of apoptosis was delayed (data not shown). Therefore, we chose RKO cells for the following experiments. To further prove caspase involved in UTD1-mediated apoptosis, we examined activation of caspase-3 and PARP by immunofluorescence staining and Western blotting. Activation of caspase-3 was confirmed by immunofluorescence, in which only cleaved caspase-3 was stained (Fig. 3D). Immunoblots of whole-cell lysates showed typical 89-kDa products of PARP cleavage as early as 24 h, accompanied by decrease of un-cleaved form of PARP (Fig. 3E top). Pretreatment with Z-VAD-FMK could reduce PARP cleavage and restore the level of un-cleaved PARP (Fig. 3E bottom). TEM also demonstrated that RKO cells underwent apoptosis after being exposed to UTD1. Cells showed typical morphological changes of apoptosis (Fig. 3F). After 12 h, cytoplasm density increased, nucleus concentrated, and nuclear membrane broke. 48 h later, vesicle formed, and eventually cells dissolved. To compare the effect of UTD1 with paclitaxel, 1 µg/ml paclitaxel was used in RKO cells. Within 36 h, UTD1 caused a higher proportion of cell death compared with paclitaxel (Fig. 3G). Importantly, UTD1 also induced more apoptosis in HCT15 cells (Fig. 3H). All results together suggested that UTD1 blocked CRC cell growth and survival via induction of apoptosis and was more effective than paclitaxel.

### UTD1 was more effective than 5-FU in vitro

**Fig. 4:**
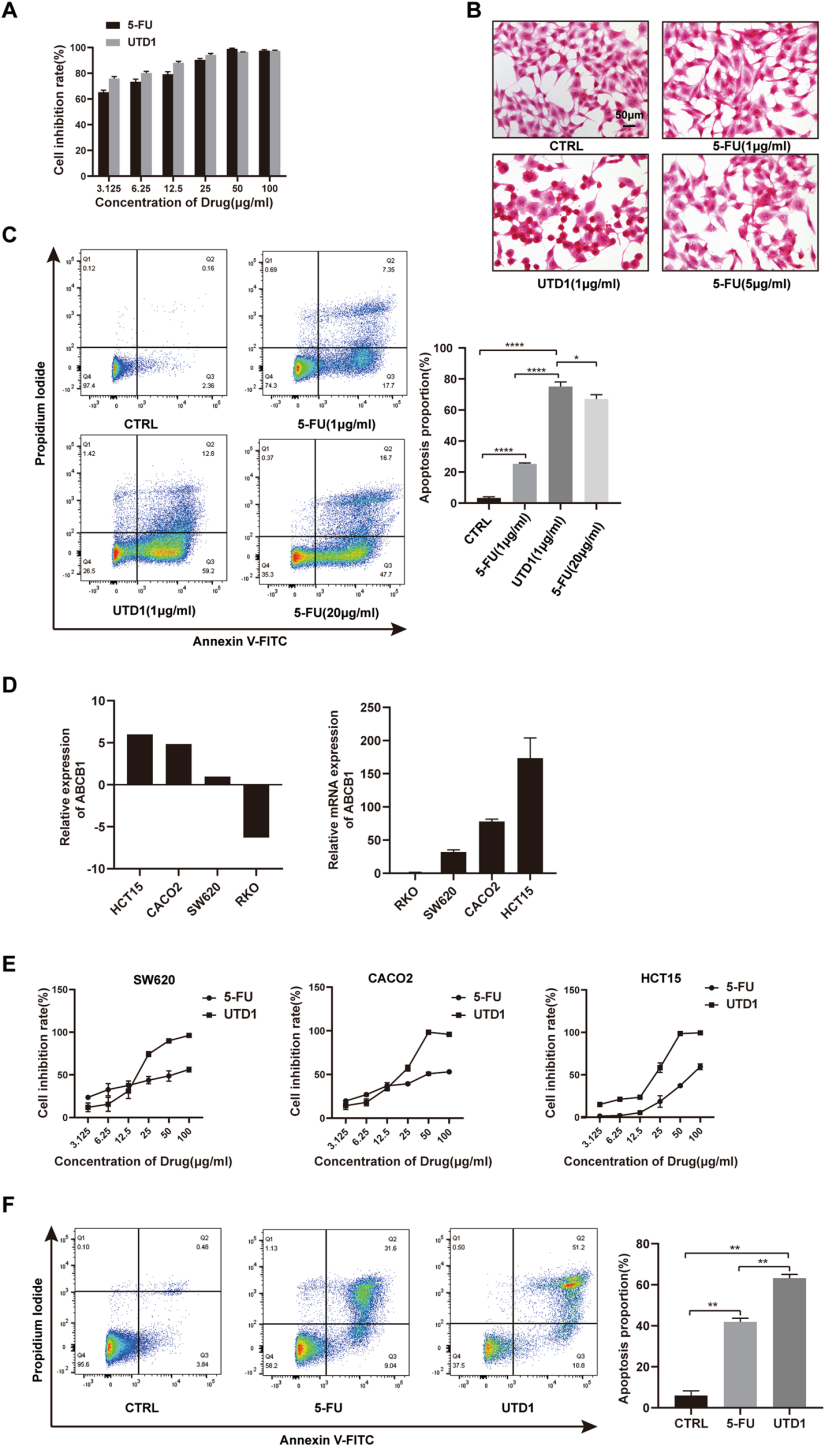
UTD1 was more effective than 5-FU in vitro. **A** RKO cells were cultured with different concentrations of UTD1 and 5-FU for 72 h. Cell viability was measured by CCK-8. **B** Morphology of RKO cells treated with 1 µg/ml UTD1 and 1 µg/ml, 5 µg/ml 5-FU for 12 h. Scale bar = 50 μm. **C** Annexin V-FITC/PI staining showed UTD1 and 5-FU induced apoptosis in RKO cells. **D** CCLE database and qRT-PCR showed expression of ABCB1 in RKO, SW620, CACO2, and HCT15 cells. **E** SW620, CACO2, and HCT15 cells were treated with different concentrations of UTD1 and 5-FU, and cell viability was measured by CCK-8. **F** After being treated with 50 µg/ml UTD1 or 5-FU for 36 h, flow cytometry was performed to test apoptosis of HCT15 cells. All experiments were performed in triplicate. Results were presented as mean ± SD, *p < 0.05, **p < 0.01, ****p < 0.0005 vs. control group.

In clinical practice, 5-FU was the first-line chemotherapy for colorectal cancer. Therefore, antitumor activities of both UTD1 and 5-FU were compared. CCK-8 showed UTD1 and 5-FU could significantly inhibit growth in a dose-dependent manner in RKO cells (Fig. 4A). RKO cells exposed to UTD1 and 5-FU exhibited morphological changes (Fig. 4B). And morphology changed more drastically in UTD1-treated condition. Flow cytometry result also showed about 72% RKO cells underwent apoptosis after being exposed to 1 µg/ml UTD1 36 h, while only 25% in 1 µg/ml 5-FU. Furthermore, 1 µg/ml UTD1 was even more effective than 20 µg/ml 5-FU(Fig. 4C). ABCB1 is one of the mechanisms that induce tumor resistance, and it is usual high-expression in colorectal cancer. To study antitumor activities of UTD1 and 5-FU in cells with different levels of ABCB1, the CCLE database was used and results were verified by qRT-PCR using RKO cells as standard (Fig. 4D). CCK-8 suggested after UTD1 treatment, IC50 of SW620, CACO2, and HCT15 cells were 16.29, 17.25, and 18.88 µg/ml, respectively, which were 51.45, 59.61, and 74.31 µg/ml after 5-FU exposure (Fig. 4E). Flow cytometry result further indicated that there were more HCT15 cells undergoing apoptosis after being treated with UTD1 (Fig. 4F). All results suggested that UTD1 was more effective than 5-FU.

### UTD1 activated ROS/JNK pathway and induced apoptosis through mitochondrial-dependent pathway

**Fig. 5:**
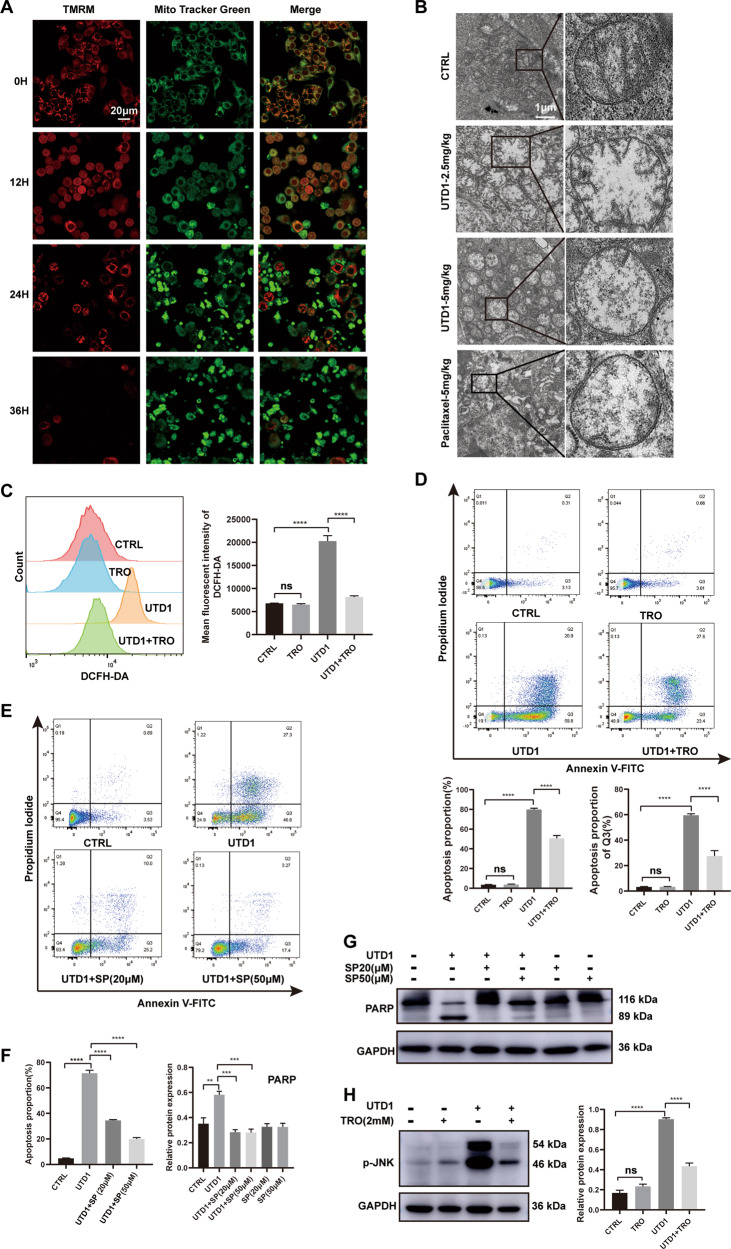
UTD1 activated ROS/JNK signaling pathway and induced mitochondrial-dependent apoptosis. **A** Confocal microscopy showed mitochondrial membrane potential decreased after RKO cells were treated with UTD1. Scale bar = 20 μm. **B** TEM demonstrated morphology changes of mitochondria in tumor tissues which were treated with UTD1 or paclitaxel. Scale bar = 1 μm. **C** Flow cytometry showed ROS increased after incubation with UTD1, and it could be inhibited by Trolox. **D** Annexin V-FITC/PI staining showed pretreatment with Trolox 2 h could decrease apoptosis. **E** JNK inhibitor, SP600125, incubated with RKO cells 2 h before UTD1 added. Flow cytometry showed apoptosis was inhibited. **F** Statistical analysis of **E** and **G**. **G** Western blotting showed SP600125 could inhibit apoptosis. **H** Antioxidant Trolox inhibited p-JNK induced by UTD1. All experiments were performed in triplicate. Results were presented as mean ± SD. ns: no statistically significant difference, **p < 0.01, ***p < 0.001, ****p < 0.0005 vs. control group.

To detect pathway causing apoptosis, mitochondrial membrane potential was measured by TMRM/Mito Tracker Green. Mitochondrial membrane potential decreased in a time-dependent manner (Fig. 5A), and it was manifested by decrease of TREM and increase of Mito Tracker Green. It suggested UTD1 induced cell apoptosis through mitochondrial pathway. After administration of UTD1 or paclitaxel, TEM further showed that mitochondrial cristae dissolved and disappeared (Fig. 5B). To investigate whether ROS was enhanced because of UDT1 treatment, DCFH-DA was used. Cells treated with UTD1 exhibited dramatic enhancement in DCFH-DA fluorescent signal, and it could be scavenged by the antioxidant and ROS scavenger, Trolox (Fig. 5C). In agreement with DCFH-DA flow cytometry, annexin V-FITC/PI staining demonstrated that apoptosis induced by UTD1 could be strongly inhibited by Trolox, especially early apoptosis (Fig. 5D). To gain further insight into the molecular mechanism of antitumor activity of UTD1, we investigated the effect of UDT1 on JNK pathway. Apoptosis of RKO cells could be decreased by JNK inhibitor, SP600125 (Fig. 5E, F left). The result of Western blotting further suggested that JNK was involved in apoptosis (Fig. 5G, F right). In addition, pretreatment with Trolox significantly reversed the phosphorylation of JNK (Fig. 5H), but production of ROS was not affected by SP600125 (data not shown). It suggested JNK activation was post ROS production. These results indicated UTD1 activated ROS/JNK pathway, consequently induced cell apoptosis through mitochondrial-dependent pathway.

### UTD1 affected mitochondrial dynamics

**Fig. 6:**
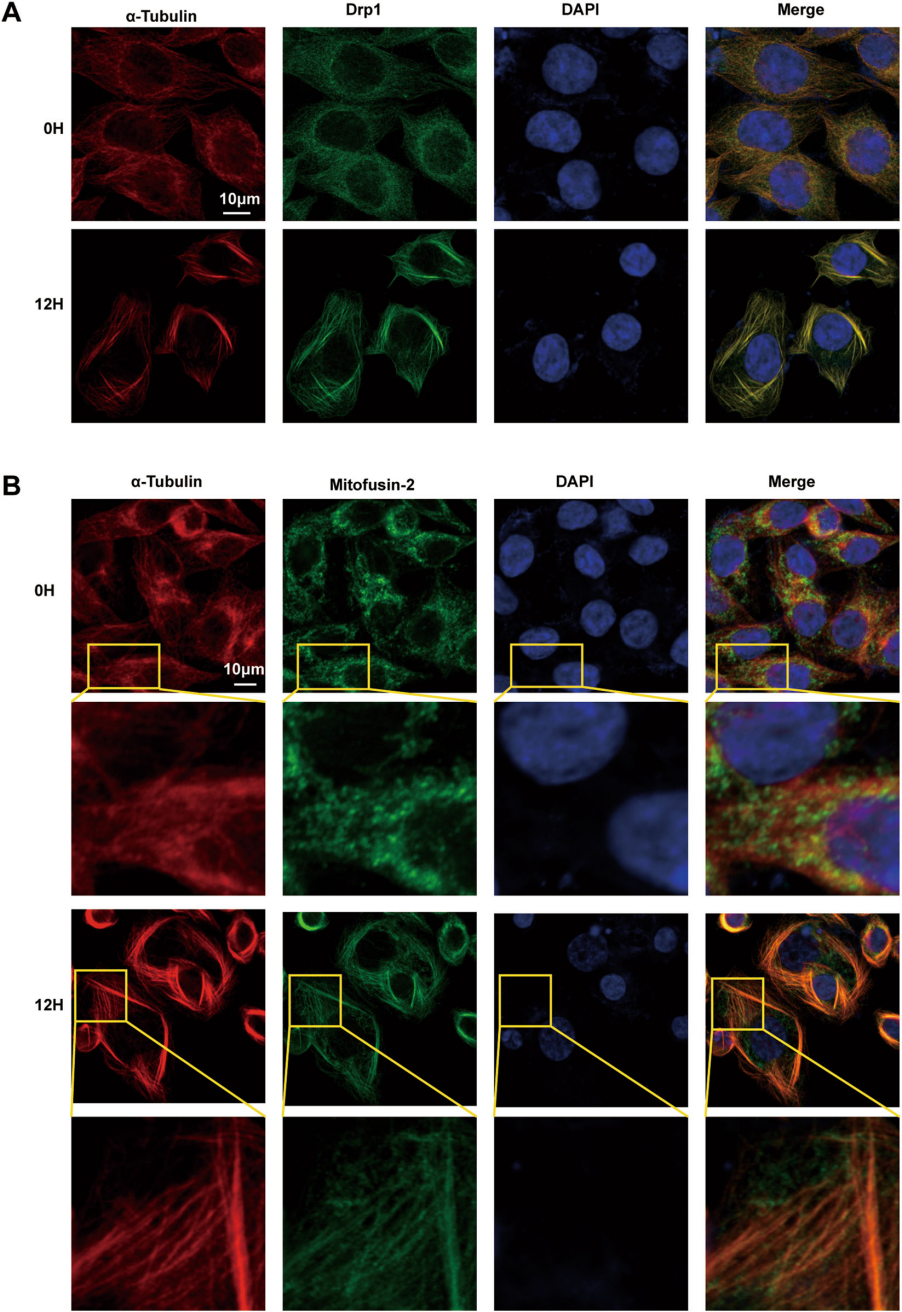
UTD1 affected mitochondrial dynamics. **A** Immunofluorescence indicated morphological dynamin-related protein 1, Drp1 changed after RKO cells were cultured with UTD1. Scale bar = 10 μm. **B** Immunofluorescence showed that UTD1 affected morphological fusion protein, Mitofusin2(Mfn2). Scale bar = 10 μm. All experiments were performed in triplicate.

Mitochondrial dynamics involves mitochondrial fusion and fission. To detect the effect of UTD1 on mitochondrial dynamics, immunofluorescence staining was used. After exposure to UTD1, mitochondrial fission protein, Drp1 almost coincided with tubulin (Fig. 6A). A similar phenomenon was found in mitochondrial fusion protein, Mitofusin-2 (Fig. 6B). These data suggested that UTD1 might affect mitochondrial dynamics, and eventually result in cell death.

### UTD1 inhibited tumor growth in RKO cell xenograft model and was more effective than paclitaxel and 5-FU

**Fig. 7:**
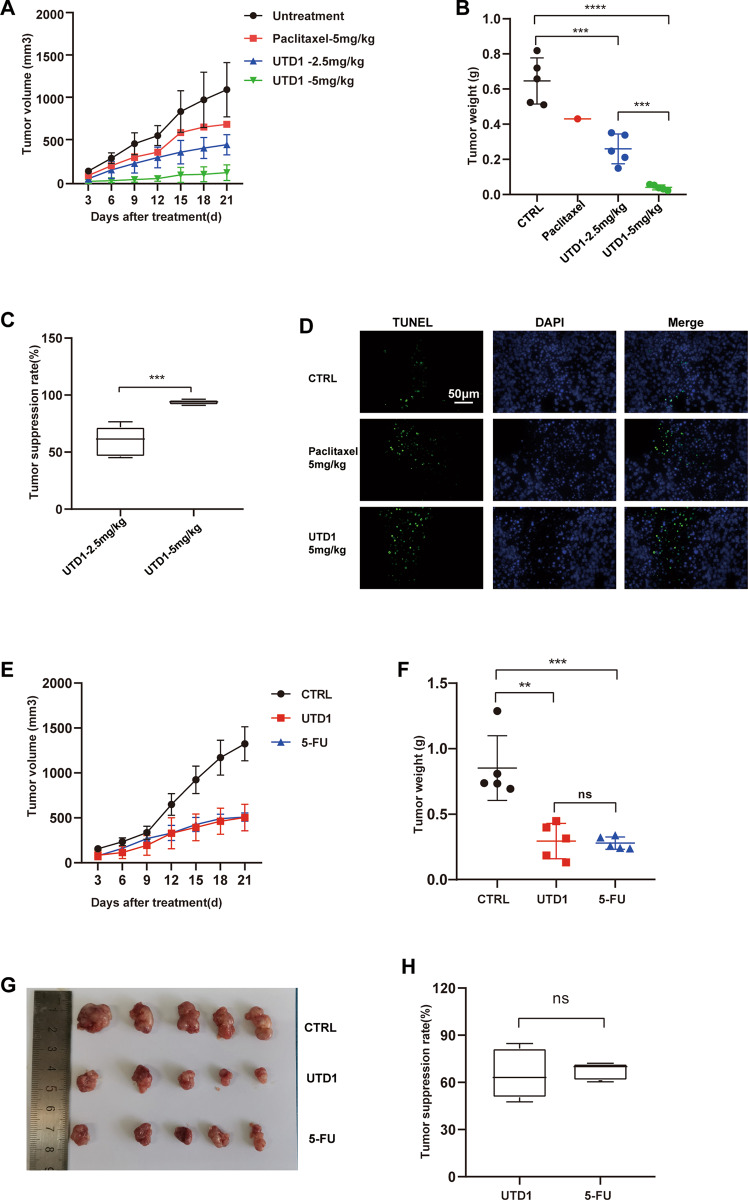
UTD1 suppressed growth of RKO cell xenograft tumor and was more effective than paclitaxel and 5-FU. **A** Changes in tumor volume during UTD1 and paclitaxel administration. **B** Tumor weights at the end of the experiment. **C** Tumor suppression rates of 2.5 and 5 mg/kg UTD1. **D** TUNEL staining of tumor tissue. Scale bar = 50μm. **E** Changes in tumor volume during UTD1 and 5-FU administration. **F** Tumor weights at the end of the experiment. **G** Image of xenograft tumor. **H** Tumor suppression rates of 2.5 mg/kg UTD1 and 25 mg/kg 5-FU. Results were presented as mean ± SD, ns: no statistically significant difference, **p < 0.01, ***p < 0.001, ****p < 0.0005 vs. control group.

To test the antitumor activity of UTD1 in vivo, RKO cell xenograft model was established. Compared with the control group, intraperitoneal administration of UTD1 significantly decreased the volume and weight of tumor in a dose-dependent manner (Figs. 7A, B, S2A). Tumor suppression rates were 62.73 and 93.63% in 2.5 mg/kg and 5 mg/kg UTD1, respectively (Fig. 7C). Compared with 5 mg/kg paclitaxel, 5 mg/kg UTD1 could significantly inhibit tumor growth. And 2.5 mg/kg UTD1 was as effective as 25 mg/kg 5-FU (Figs. 7E, F, G, S2C). There was no statistical significance between 2.5 mg/kg UTD1 and 25 mg/kg 5-FU in tumor suppression rate (Fig. 7H), probably suggesting that UTD1 was more effective than 5-FU. TUNEL-positive cells increased after UTD1 treatment (Fig. 7D). IHC showed that as UTD1 increased, expression of Ki-67 decreased, whereas p-JNK and Cytochrome C increased. UTD1 group increased more than paclitaxel group (Fig. S2B). These results suggested UTD1 could inhibit tumor growth in RKO cell xenograft model and was more effective than paclitaxel and 5-FU.

### UTD1 was safer than paclitaxel and 5-FU in vivo

**Fig. 8:**
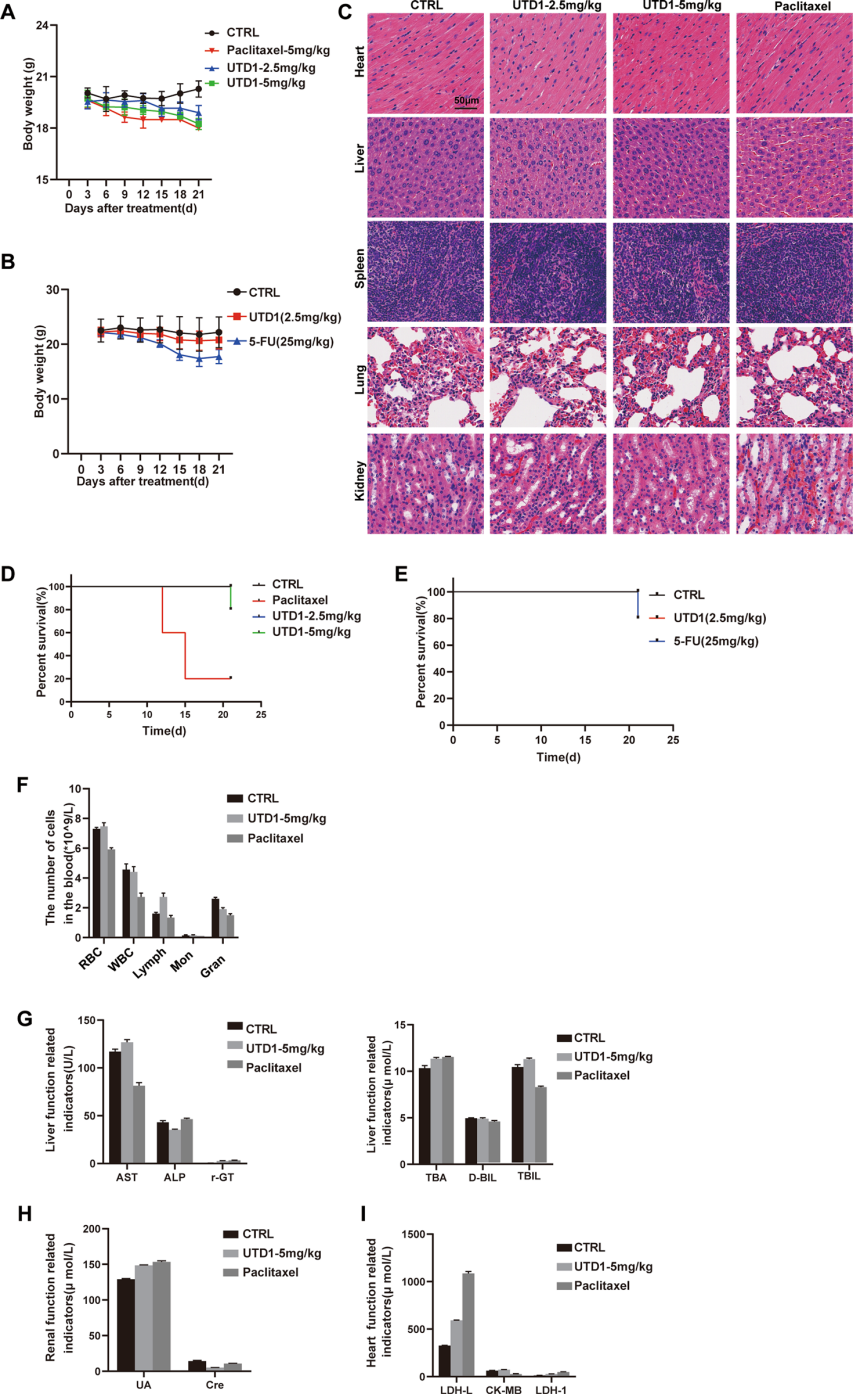
UTD1 was safer than paclitaxel and 5-FU in vivo. **A**, **B** Changes in body weight during drug administration. **C** H&E staining of heart, liver, spleen, lung, and kidney in tumor specimens. Scale bar = 50 μm. **D**, **E**. Survival curve of nude mice during drug administration. **F** Whole blood cell analysis of mice after UTD1 and paclitaxel administration. **G** Evaluation of liver function in mice. **H** Evaluation of renal function in mice. **I** Evaluation of heart function in mice. Results were presented as mean ± SD.

We further determined the safety of UTD1 in animal model. During 20 days’ administration of drugs, little weight loss was observed in UTD1 group compared with paclitaxel and 5-FU groups (Fig. 8A, B). Furthermore, no significant changes were observed in the colors and textures of vital organs, including heart, liver, spleen, lung, and kidney (Fig. 8C). Survival rate of 5 mg/kg paclitaxel was 20%, 25 mg/kg 5-FU was 80%, 5 mg/kg UTD1 was 80%, and 2.5 mg/kg UTD1 was 100% (Fig. 8D, E). Whole blood cell analysis indicated blood cell count decreased less than paclitaxel after UTD1 treatment (Fig. 8F). The indexes of liver, kidney, and heart function showed 5 mg/kg UTD1 group was closer to control group, although they were all within normal range (Fig. 8G, H, I). These results confirmed that UTD1 2.5 mg/kg and even 5 mg/kg did not cause obvious systemic toxicity in vivo. Under the same conditions, UTD1 was safer than paclitaxel. And with same antitumor effect, UTD1 was needed less than 5-FU; thus it had fewer side effects.

## Discussion

Paclitaxel is widely used in treating varieties of solid tumors. But it failed in CRC clinical trials because of side effects or multidrug resistance^22,23^.

In this study, we investigated antitumor activity of UTD1 compared with paclitaxel and 5-FU in human CRC cells and explored underlying molecular mechanisms. Our results showed that UTD1 inhibited cell proliferation and colony formation, altered microtubule dynamics, blocked cells at G2/M phase followed by apoptosis. Importantly, UTD1 was more effective than paclitaxel and 5-FU, especially in ABCB1 high-expression cells, such as HCT15 cells.

Microtubule-stabilizing agents are known to cause microtubule polymerization^31,32^. As one of microtubule-stabilizing agents, effect of UTD1 on microtubule was consistent with previous studies^33,34^. After UTD1 exposure, microtubule bundles and asters formed. Anti-microtubule agents arrested cells in G2/M phase^35–37^. In our research, flow cytometry revealed that UTD1 could induce cell cycle G2/M block in a time-dependent manner.

Li et al.^38–40^ have reported after being blocked by anti-mitotic drugs for a long time that cells underwent apoptosis mainly through mitochondrial pathway. Our findings clearly demonstrated that mitochondrial membrane potential was decreased, and caspase-3 and PARP were cleaved after UTD1 incubation. UTD1 treatment increased the level of ROS, which could activate JNK, increase the phosphorylation of JNK, eventually resulting in cell death. These suggested that ROS/JNK pathway might be involved. Mitochondrial dynamics have been reported associated with cell death^41^, after exposure to UTD1, both mitochondrial fission and fusion protein, Drp1 and Mitofusin2 changed, indicating that UTD1 might affect mitochondrial dynamics, which also could trigger cell death.

Given the activity of UTD1 against CRC cells in vitro, it was important to evaluate it in preclinical CRC xenograft model. In vivo, UTD1 significantly inhibited growth of CRC. Compared with the same dose of paclitaxel, tumor volume was significantly reduced in UTD1 group. And 2.5 mg/kg UTD1 was as effective as 25 mg/kg 5-FU. Compared with four mice dead in paclitaxel group, UTD1 caused only one mouse death. And there was less body weight loss in UTD1 group compared with paclitaxel and 5-FU groups. The safety assessment of UTD1 group was closer to that of control group. These data suggested that UTD1 displayed robust antitumor activity in CRC xenograft model with controllable toxicity.

In summary, UTD1 is efficacious in suppressing the growth of CRC cells. Furthermore, it has considerable antitumor efficacy with tolerable side effects in vivo. We anticipate that UTD1 may prove to be a promising agent in CRC therapy.

## Materials and methods

### Cell lines and culture

Human CRC cell lines RKO, HCT116, CACO2, SW620, and HCT15 were purchased from Cell Culture Center of Institute of Basic Medical Sciences, Chinese Academy of Medical Sciences. Cells were cultured in RPMI-1640 medium with 10% Fetal Bovine Serum (FBS) and 1% penicillin and streptomycin (Gibco), and incubated at 37 °C under an atmosphere of 95% air and 5% CO_2_.

### Materials

In vitro, UTD1, paclitaxel, and 5-Fluorouracil (5-FU) (stock solution 10 mg/ml) were diluted in complete medium according to required concentration before use. Complete medium was used as control. In vivo, drugs were diluted in 0.9% sodium chloride aqueous solution before administration. And 0.9% sodium chloride aqueous solution was used as control. RPMI-1640 medium, FBS, and crystal violet were purchased from SIGMA. PI/RNase Staining Buffer and FITC Annexin V Apoptosis Detection Kit were purchased from BD Biosciences. Paclitaxel, 5-FU, Z-VAD-FMK, SP600125, and Trolox were purchased from Med Chem Express (Shanghai, China). Antibodies against cyclinB1, CDC2, P21, PARP, Cleaved caspase-3, cytochrome C, phospho-JNK, Ki-67, Drp1, Mitofusin-2, and secondary antibody were purchased from Cell Signaling Technology (Beverly, MA, USA). CyclinA2, glyceraldehyde-3-phosphate dehydrogenase (GAPDH), α-Tubulin, and secondary antibody were purchased from Abcam (UK).

### Cell proliferation assay

Cell proliferation activity was detected using Cell Counting Kit-8 (CCK-8) (Dojindo, Japan). RKO, HCT116, SW620, CACO2, and HCT15 cells were seeded in a 96-well plate. Different concentrations of UTD1 and 5-FU were added and incubated for 72 hour (h) or 1 μg/ml UTD1 was added and incubated for different time-points. According to the protocol provided, CCK-8 working solution was added and incubated for 3 h at 37 °C; absorbance was measured at 450 nm with a microplate reader (PerKinElmer). IC50 was calculated using GraphPad Prism8.

### Cell morphology

To investigate the effect of UTD1 and 5-FU on cell morphology, cells were seeded in a 6-well plate with cover slips at a density of 2 × 10^5^/ml. UTD1 and 5-FU were added and incubated. Cells were fixed with 95% ethanol for 15 min, stained with hematoxylin and eosin (HE). Cell morphology changes were observed with microscopy (Leica DM6 B).

### Colony formation assay

Crystal violet was used to detect colony formation of cells. After being incubated with UTD1, 1000 cells/well were seeded in a 6-well plate. Medium was changed in 2–3 days and incubated for 14 days. The colonies were fixed with 100% methanol and stained with 0.5% crystal violet. The numbers of colony were counted by microscopy (OLYMPUS CKX53).

### Immunofluorescence staining assay

Microtubule morphology changes, apoptosis, and mitochondrial changes were observed by immunofluorescence staining assay. Cells (3 × 10^3^ cells/well) were dispensed in chamber on poly-D-lysine-coated eight-chamber microscope slides. After treatment, cells were fixed with 4% paraformaldehyde for 20 min and blocked with 5% bovine serum albumin-0.3% Triton X-100 in phosphate buffered saline (PBS) for 1 h at room temperature. Cells were incubated with primary antibody at 4 °C overnight. Antibodies used were as follows: α-tubulin, cleaved caspase-3, Drp1, mitofusin-2, followed by incubating with secondary antibody. Samples were mounted with mounting medium with DAPI (Abcam). Images were acquired with a confocal laser scanning microscope (Zeiss LSM880).

### Cell-cycle analysis

To study cell cycle distribution, cells were cultured with UTD1, collected and fixed with 70% ethanol at −20 °C for more than 24 h. 500 μl PI/RNase staining buffer was added and incubated for 15 min at room temperature protected from light. Flow cytometry was performed (BD LSRFortessa) and data were analyzed by FlowJoV10.

### Apoptosis analysis

After incubation with drugs, cells were collected and resuspended in 100 μl of 1× binding buffer containing 5 μl fluorescent-labeled Annexin V and 5 μl propidium iodide (PI), incubated for 15 min in dark. Samples were analyzed by flow cytometry and data were analyzed by FlowJoV10.

### Western blotting analysis

Proteins are detected by Western blotting. After incubation, cell lysates were heated in 1x SDS loading buffer for 15 min at 100 °C. Proteins were separated by SDS-PAGE gel, after being transfered to 0.45 μm polyvinylidene fluoride membranes, blocked with 5% BSA for 1 h. Membranes were incubated with antibodies against cyclinB1, cyclinA2, CDC2, P21, PARP, p-JNK, and GAPDH at 4 °C overnight. Secondary antibody was incubated for 1 h. Bands were detected by a chemiluminescence imaging system (Amersham Imager 600) with Super ECL Detection Reagent (Yeasen, Shanghai, China). Results were analyzed by Image J.

### Quantitative Real-Time Polymerase Chain Reaction (qRT-PCR)

Total RNA was extracted from RKO, SW620, CACO2, and HCT15 cells using TRIzol reagent (Invitrogen, USA). The cDNA was synthesized with PrimeScript™ RT Master Mix (Takara, Japan). qRT-PCR was carried out on LightCycler480 System (Roche, Switzerland) using TB Green™ Premix Ex Taq ™ (Takara). The primers are as follows: ABCB1:5′-GATTGCTCACCGCCTGTCCAC-3′ and 5′-CGTGCCATGCTCCTTGACTCTG-3′; GAPDH:5′-ACATCGCTCAGACACCATGG-3′ and 5′-ACCAGAGTTAAAAGCAGCCCT-3′.

### Transmission Electron Microscope (TEM)

After treatment, RKO cells or tumor tissues were fixed with 2.5% glutaraldehyde at 4 °C for 1 h, 1% osmic acid was added at 4 °C for another 1 h. After alcohol dehydration, samples were infiltrated for 1.5 h and immersed in pure epon812 overnight. Then they were polymerized at 65 °C for 2 days. Samples were observed and imaged using TEM (Talos L120C).

### Mitochondrial membrane potential analysis

Tetramethylrhodamine (TMRM) and Mito Tracker Green (Invitrogen) were used to monitor mitochondrial in RKO cells. After being treated, cells were incubated with TMRM and Mito Tracker Green at 37 °C for 30 min. Cells were washed with medium and analyzed with a confocal laser scanning microscope.

### Measurement of ROS generation

Reactive Oxygen Species Assay Kit (Beyotime, Shanghai, China) was used to detect intracellular ROS generation. RKO cells were exposed to UTD1 with or without Trolox. Cells were stained with 10 μM DCFH-DA at 37 °C for 20 min. The level of ROS was measured by flow cytometry.

### In vivo xenograft mice experiment

All animal experiments were conducted according to the principles of the Institutional Animal Care and Use Committee at Shanghai Jiaotong University. Six-week BALB/c-nu female mice (Shanghai Slac Laboratory Animal Co., Ltd., Shanghai, China) weighing approximately 20 g were introduced to establish xenograft tumor model. 4 × 10^6^ RKO cells were subcutaneously injected into right flank of mice in 200 μl RPMI-1640 medium. When tumor volume reached about 100 mm^3^, mice were randomly assigned to different groups (*n* = 5) and treated every two days for 20 days. 4 groups were treated with UTD1 (2.5 mg/kg and 5 mg/kg, ip), paclitaxel 5 mg/kg (ip) or vehicle control. Three groups were treated with UTD1(2.5 mg/kg, ip), 5-FU (25 mg/kg, ip), or vehicle control. Tumor size was measured and mice were weighed every three days. Tumor volume was calculated by the formula: *V* = 0.5 × *a* × *b*^2^, where *a* was the length (mm), *b* was the width (mm), and *V* was the volume of tumor. At the end of the experiment, all mice were killed and weights of tumor were recorded. To assess anticancer activity of drugs, percentage of inhibition was calculated as following: Inhibition% = (1−mean of tumor weight of treated group/mean of tumor weight of control group) × 100%.

### Immunohistochemistry (IHC) analysis

Immunohistochemical staining for p-JNK, Ki-67, and Cytochrome C was performed according to the manufacturer’s instruction. Briefly, sections were incubated with blocking solution for 1 h at room temperature, followed by overnight incubation with primary antibodies at 4 °C. After being incubated with horse anti-Rabbit IG for 2 h at room temperature and mounted, specimens were observed under microscopy.

### Statistical analysis

All results are given as mean ± SD. Statistical analysis was performed using GraphPad Prism 8. One-way ANOVA and unpaired Student’s *t* test were used to calculate differences among various experimental groups. *P*-values < 0.05 were considered statistically significant.

## Supplementary information

Figure S1

Figure S2

Supplementary figure legends
