# Supplementary figures and images for: Utidelone inhibits growth of colorectal cancer cells through ROS/JNK signaling pathway
Source: Cell Death Dis. 2021 Apr 1;12(4):338. doi: 10.1038/s41419-021-03619-6 (PMC8016927; doi:10.1038/s41419-021-03619-6)

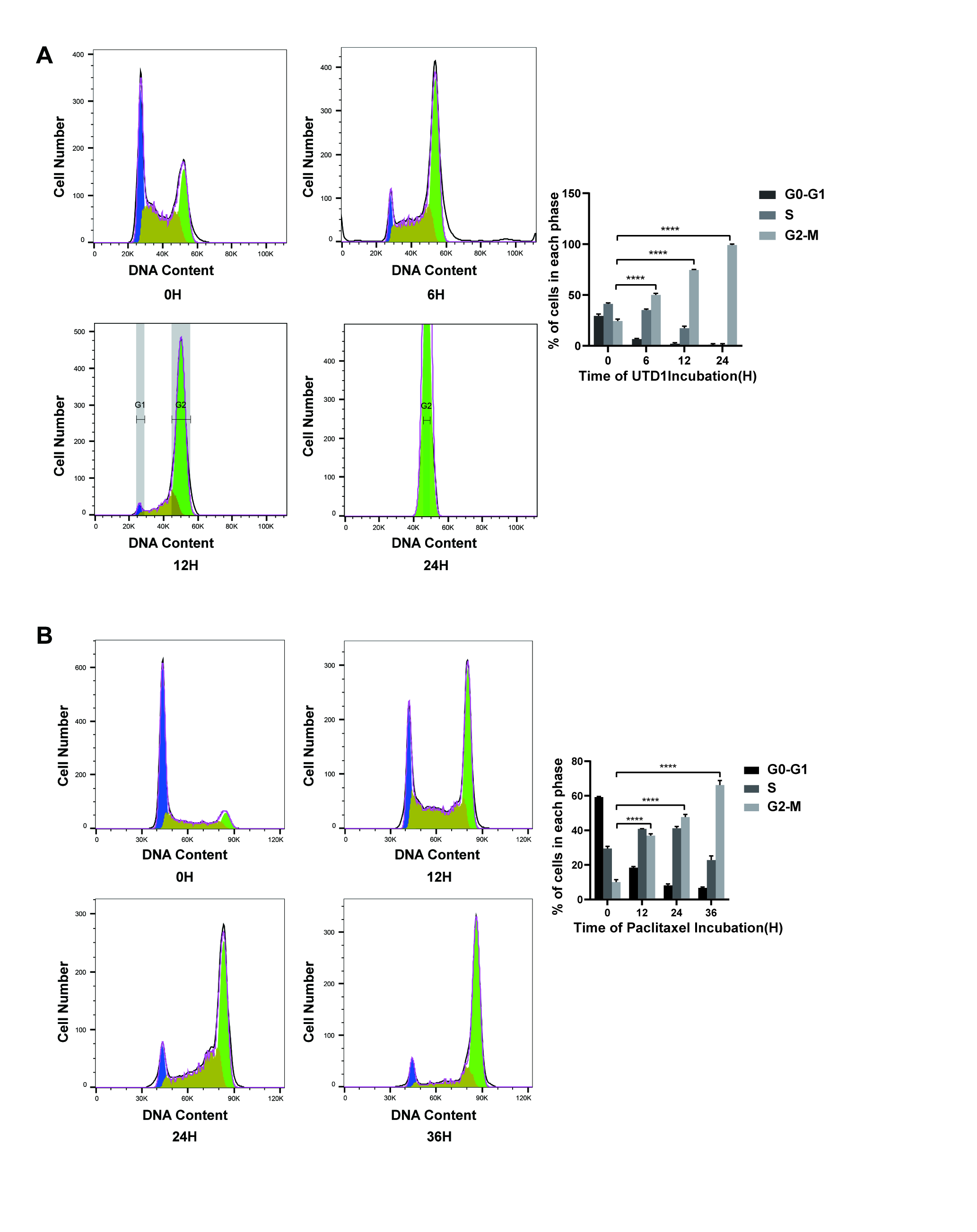

Supplement: Supplementary file 1 — Figure S1 [file 41419_2021_3619_MOESM1_ESM.tif]

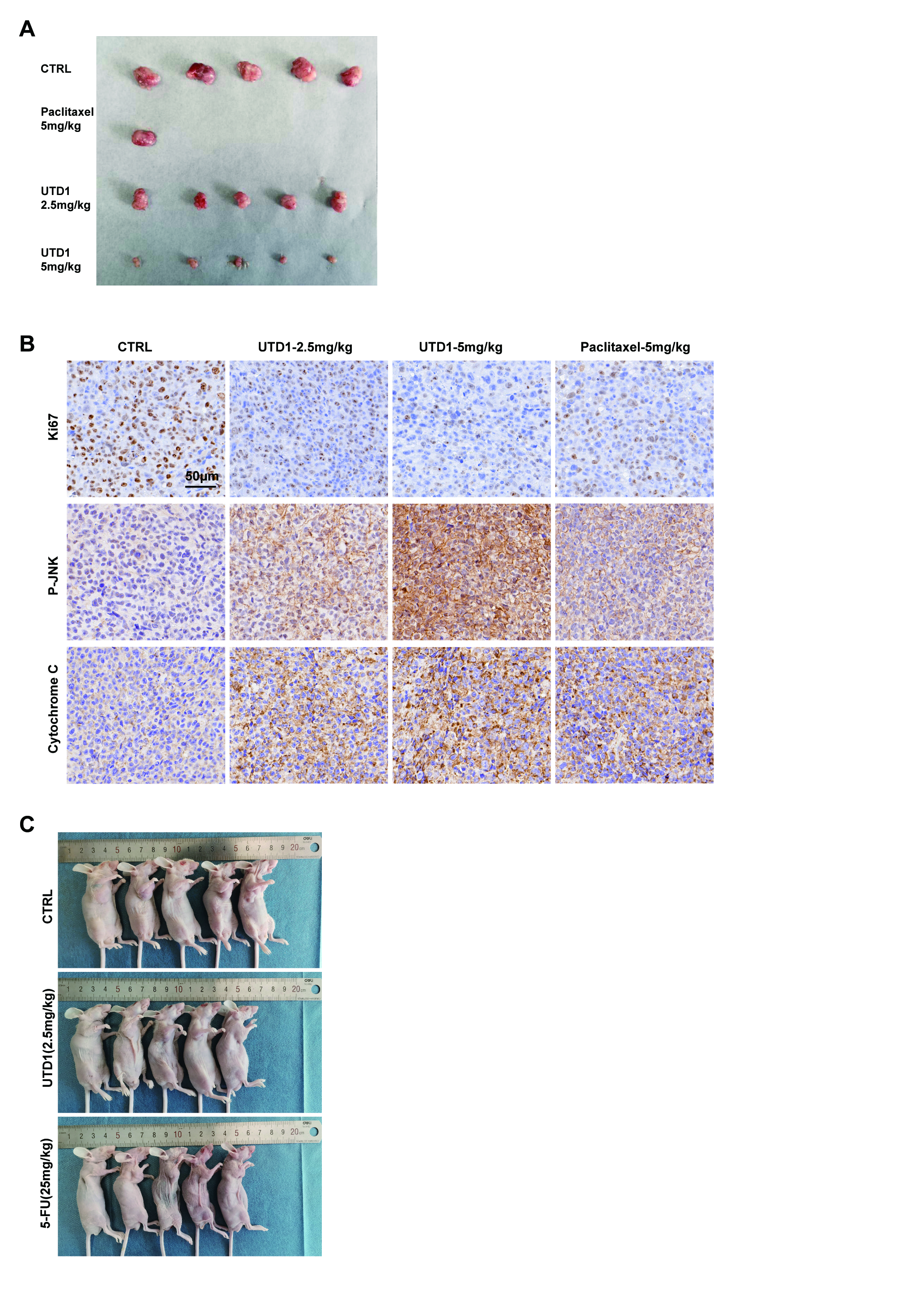

Supplement: Supplementary file 2 — Figure S2 [file 41419_2021_3619_MOESM2_ESM.tif]
