# Supplementary material for: Utidelone inhibits growth of colorectal cancer cells through ROS/JNK signaling pathway
Source: Cell Death Dis. 2021 Apr 1;12(4):338. doi: 10.1038/s41419-021-03619-6 (PMC8016927; doi:10.1038/s41419-021-03619-6)
Supplement: Supplementary file 3 — Supplementary figure legends [file 41419_2021_3619_MOESM3_ESM.docx]

**Figure legends**

**Fig.S1.** **UTD1 Induced CRC Cells G2/M Phase Arrest as Paclitaxel.** (A). Flow cytometry result showed UTD1 arrested HCT116 cells in G2/M phase. (B). Flow cytometry result showed paclitaxel arrested RKO cells in G2/M phase. All experiments were performed in triplicate. Results were presented as mean ± SD, ****p<0.0005 vs. control group.

**Fig.S2. UTD1 Suppressed Growth of RKO Cell Xenograft Tumor and Was More Effective than Paclitaxel and 5-FU.** (A). Image of xenograft tumor. (B). Immunohistochemical staining of tumor specimens. Scale bar =50μm. (C). Image of tumor xenograft model mice.
